# Evaluating Clinical Pharmacist Interventions in a Tertiary Care Hospital: A Retrospective Study from Saudi Arabia

**DOI:** 10.3390/healthcare13192504

**Published:** 2025-10-02

**Authors:** Abdulhamid Althagafi

**Affiliations:** Department of Pharmacy Practice, Faculty of Pharmacy, King Abdulaziz University, Jeddah P.O. Box 22252, Saudi Arabia; aalthagfi@kau.edu.sa; Tel.: +966-126400000/21146; Fax: +966-126951696

**Keywords:** clinical pharmacy services, medication safety, pharmacist interventions, patient outcomes, dosage errors

## Abstract

**Background**: Clinical pharmacy services (CPSs) play a key role in ensuring medication safety, optimizing pharmacotherapy, and improving patient outcomes. While their benefits are well-documented globally, their specific impact within the Saudi healthcare system remains underexplored. **Objective**: This study aimed to evaluate the impact of pharmacist-led interventions in a tertiary medical center in Saudi Arabia. Methods: A retrospective chart review was conducted at a 1200-bed academic hospital in western Saudi Arabia. Pharmacist interventions documented between 1 January 2023 and 31 December 2023 were analyzed. Interventions were categorized into 13 types, including dosage errors, unavailable medications, and drug–drug interactions. Descriptive statistics were used to summarize the data. **Results**: A total of 38,143 pharmacist interventions were recorded. Dosage errors accounted for 77.2% (n = 29,584) of interventions, followed by issues with medication availability (6.57%, n = 2519) and incorrect medication orders (4.59%, n = 1761). The most frequently implicated medications were acetylsalicylic acid, enoxaparin, and paracetamol, collectively representing 43.55% of interventions. The highest intervention rates were in the Emergency Department (25.3%, n = 11,050), Oncology Clinics (9.81%, n = 4285), and Male Medical Units (9.43%, n = 4119). **Conclusions**: Clinical pharmacists play a significant role in reducing medication errors and improving patient safety across various specialties. Their targeted interventions optimize therapeutic outcomes, highlighting the need for integrating advanced tools and expanding CPSs to meet evolving healthcare demands in Saudi Arabia.

## 1. Introduction

Clinical pharmacy services (CPSs) are an integral part of global healthcare systems, aimed at ensuring safe, effective, and efficient use of medications. These services encompass a wide range of activities, including optimizing pharmacotherapy, resolving drug-related problems, and enhancing patient outcomes through pharmacist-led interventions [1]. Over the decades, CPS has undergone a significant transformation, evolving from traditional roles focused primarily on dispensing to integral components of multidisciplinary healthcare teams [2]. This evolution reflects the growing recognition of pharmacists as essential healthcare providers, whose contributions directly impact clinical and economic outcomes [3].

Globally, pharmacist-led interventions have demonstrated not only improvements in clinical outcomes such as reduced medication errors and enhanced adherence, but also substantial benefits by minimizing adverse events and optimizing resource utilization, as seen in both inpatient and outpatient settings [4,5,6,7]. These benefits highlight the crucial role of pharmacists in promoting medication safety and enhancing overall health outcomes. In Saudi Arabia, CPS has gained prominence as part of the nation’s ongoing healthcare reforms. Initiatives driven by the Ministry of Health have emphasized the integration of pharmacists into multidisciplinary teams to enhance medication safety and therapeutic outcomes [8].

National studies have shown that clinical pharmacy interventions significantly reduce medication errors, improve adherence to evidence-based guidelines, and contribute to improved disease management. This shift aligns with Saudi Vision 2030, which prioritizes healthcare transformation through improved quality, efficiency, and patient-centered care. However, challenges in medication management persist, both globally and locally. These challenges include the growing complexity of therapeutic regimens, the increasing prevalence of chronic diseases, and variability in healthcare system capacities. In Saudi Arabia, additional barriers, such as limited access to resources, uneven distribution of clinical pharmacists, and variability in the implementation of CPS across institutions, further complicate efforts to optimize medication use [9].

Addressing these issues requires a systematic evaluation of CPS to identify areas for improvement and to inform targeted interventions. The rationale for this study stems from the critical need to evaluate and enhance CPS in specific healthcare contexts. While international studies have extensively documented the benefits of CPSs, there is a paucity of large-scale, multicenter studies in Saudi Arabia that evaluate their effectiveness using real-world data on medication error types, high-risk drugs, and departmental patterns, limiting localized policy guidance. Detailed analyses of pharmacist-led interventions are essential for understanding their scope, identifying trends, and providing evidence to guide policy and practice improvements. International studies have investigated the impact of clinical pharmacy interventions and their outcomes on health-related quality of life, as well as evaluated the role of critical care pharmacy service interventions in preventing or minimizing drug-related problems [10,11]. This study aimed to evaluate the frequency and type of pharmacist-led interventions in a tertiary medical center in Saudi Arabia.

## 2. Methods

### 2.1. Settings

This retrospective chart review was conducted at a tertiary medical center, one of the largest academic hospitals in the western region, with a capacity of 1200 beds and multi-specialty clinics. The study analyzed pharmacist interventions from 1 January 2023 to 31 December 2023. Medication orders were electronically sent to the pharmacy via electronic health records. A licensed pharmacist then verified the medication orders before dispensing and administration. At the time of this study, we had four different clinical pharmacy specialties: medical ICU, surgical ICU, pediatric ICU, and nephrology pharmacists. The most common interventions by medication type and specialties requiring the most frequent interventions were also evaluated. Clinical pharmacists have documented their interventions and therapeutic drug monitoring activities across specialties.

### 2.2. Data Collection

We collected all data pertinent to our study, including the name of the medications ordered, dosage form, frequency, type of intervention, medical ward in which the medication order was written, the medical team that ordered the medication, the clinical pharmacy specialty progress note, and the patient’s condition under the clinical pharmacist’s supervision. All documented pharmacists’ interventions in our healthcare record were included in our study and categorized as follows: wrong dose, medication availability, as many treating medical teams rely on pharmacy staff for information on updated formulary, wrong medication pharmacy clarification, drug information, duplicate therapy, dosing regimen errors, drug–drug interactions, drug monitoring, and incorrect medication dosage form. The data collection process was conducted by trained pharmacy students under the supervision of academic experts. Pharmacy staff members are highly qualified professionals with extensive training and education, ranging from graduates of national and international Doctor of Pharmacy programs to postgraduate master’s degrees, PhD degrees, and residency programs.

The most common interventions by medication type and specialties requiring the most frequent interventions were also evaluated. Clinical pharmacists documented their interventions and therapeutic drug monitoring activities across specialties. Descriptive statistics are summarized using frequencies and percentages.

### 2.3. Statistical Analysis

Descriptive statistical analyses were conducted using IBM SPSS Statistics Version 31.0 (IBM Corp.; Armonk, NY, USA). Frequencies and percentages were used to summarize categorical variables such as intervention types, involved medications, and ordering specialties [12].

### 2.4. Inclusion and Exclusion Criteria

Inclusion criteria included all pharmacist-documented interventions on medication orders within the study period. Exclusion criteria included incomplete intervention records such as draft interventions and duplicated entries of pharmacy staff on the same medication orders and same interventions.

### 2.5. Definitions

Wrong dose was defined as an intervention involving the adjustment of medication strength or frequency that was inconsistent with guidelines or the patient’s condition. Wrong medication was defined as administering a medication that was not indicated or as prescribing an alternative medication option that was more effective than the one prescribed prior to the intervention. An incorrect medication dosage form was defined as an incorrect order of medication format (e.g., the medical team ordered a capsule dosage form while only the tablet format was available at the time of prescription). Pharmacy clarification was defined as clarifying requests for prescriber orders due to incomplete, ambiguous, or conflicting prescription information. Dosage regimen errors were defined as errors in the duration or timing of doses. Drug monitoring was defined as interventions related to ordering or interpreting therapeutic drug levels (e.g., vancomycin and aminoglycosides).

### 2.6. Outcomes

The primary outcome was the most frequent type of pharmacy intervention. For secondary outcomes, we aimed to evaluate the most frequent intervention by medication type, the most frequent intervention by floor/ward specialty, progress note documentation by pharmacy specialty, and patient condition.

## 3. Results

A total of 38143 pharmacist interventions were documented, with dosage errors identified as the predominant category (Table 1). Wrong dose interventions accounted for 77.2% of all cases (n = 29,584). Issues with medication availability ranked second, accounting for 6.57% (n = 2519), followed by incorrect medication administration at 4.59% (n = 1761, 95% CI: 4.45–4.74%). Pharmacy clarifications and drug information requests were recorded in 3.24% (n = 1243) and 3.23% (n = 1237) of the interventions, respectively. Additionally, drug class duplications and errors in dosing regimens, although less frequent, accounted for notable intervention rates of 2.29% (n = 878) and 1.58% (n = 609), respectively. This range of interventions highlights the diversity and complexity of pharmacists’ contributions to patient safety.

Pharmacist interventions are associated with a wide variety of medications, reflecting the extensive scope of their practice. Acetylsalicylic Acid was the most frequently used medication, accounting for 15.87% (n = 4498) of all interventions. Enoxaparin followed at 14.91% (n = 4224), while paracetamol ranked third at 12.77% (n = 3619). Other medications with high intervention rates included ondansetron (9.59%, n = 2716) and omeprazole (9.39%, n = 2660) (Table 2). These trends suggest that commonly prescribed or widely utilized medications are frequent sources of pharmacist intervention. Given the risks associated with incorrect dosing or administration of these drugs, especially anticoagulants and analgesics, pharmacist interventions likely play a key role in preventing adverse drug events (ADEs) and optimizing therapeutic outcomes.

The analysis by clinical specialty revealed that the Emergency Department accounted for the most significant proportion of pharmacist interventions (25.3%, n = 11,050). This high frequency likely reflects the fast-paced, high-risk environment of emergency care, where rapid prescription decisions may be prone to errors. Other specialties with significant intervention rates included Oncology Clinics (9.81%, n = 4285), Male Medical Units (9.43%, n = 4119), and Hematology Clinics (9.25%, n = 4041). The high rate in oncology and hematology clinics may stem from the complexity of chemotherapy regimens, where precise dosing is critical for minimizing toxicity and ensuring therapeutic efficacy. Medical Clinics also reported notable intervention activities at 8.79% (n = 3839). These findings demonstrate that pharmacist involvement is essential across a range of clinical settings, from acute care to specialized and chronic disease management (Table 3).

In our study, a total of 34,111 out of 38,143 pharmacist interventions were documented, resulting in an overall acceptance rate of 89.4%. Notably, the majority of accepted interventions involved wrong dose adjustments and medication unavailability—categories strongly associated with potential harm if left unresolved. This reinforces the trust placed in clinical pharmacists and supports their role as key contributors to medication safety and therapeutic optimization in complex healthcare environments.

A total of 729 progress notes were documented and distributed across the four key specialties. Pediatric ICU accounted for the highest proportion of progress notes (44.4%, n = 324), followed by Surgical ICU (25.4%, n = 185), Medical ICU (16.9%, n = 123), and Nephrology (13.3%, n = 97) (Table 4).

Further analysis of patient conditions revealed that most progress notes pertained to stable patients (58.7%, n = 428). Notes for patients in poor condition were fewer at 5.3% (n = 39), while improving patients and those experiencing deterioration accounted for 1.9% (n = 14) and 0.4% (n = 3), respectively. This distribution suggests that pharmacists primarily focus on optimizing therapy for chronic and stable conditions, while still playing a targeted role in acute and deteriorating cases (Table 5).

## 4. Discussion

This study demonstrates the important role of clinical pharmacists in identifying and resolving medication-related issues within tertiary medical centers, presenting findings with significant implications for healthcare systems. The predominance of dosage errors, which constituted 77.2% of all pharmacist interventions, reflects the complexity of therapeutic regimens in tertiary care and variations in prescriber adherence to dosing protocols, particularly with high-risk medications such as anticoagulants, analgesics, and nephrotoxic agents. This is consistent with Onatade et al., who reported that 35% of interventions in UK hospitals addressed dosing errors, with an overall acceptance rate of 84% by prescribers [13]. Their findings also showed that pharmacist interventions improved therapeutic outcomes and prevented potential harm in 22% of cases. Onatade et al. further emphasized the economic and organizational benefits of pharmacist engagement, including cost reduction and optimized resource use. Similarly, Ylä-Rautio et al. found that 39.2% of drug-related problems (DRPs) in community settings involved dosing uncertainties, particularly with high-risk OTC medications such as NSAIDs [14]. These findings underscore the pharmacist’s crucial role in preventing inappropriate medication use through direct patient interaction and counseling. Baptista et al. reinforced this importance by demonstrating a threefold increase in recorded interventions following the implementation of structured digital documentation [15]. In their study, 63% of interventions addressed dosing inaccuracies, resulting in a 41.4% improvement in patient care quality. Such structured systems ensure that pharmacists’ contributions are systematically recorded, enhancing governance and policy-making. These comparisons demonstrate that pharmacist integration enhances both the safety and quality of care, while also preventing adverse outcomes.

In our study, 89.4% of interventions were accepted by prescribers, reflecting strong interprofessional collaboration and trust in clinical pharmacy services. This rate is comparable to those reported in Saudi and regional studies. Althomali et al. noted a 98.5% acceptance rate in ICUs, while Alkhanbashi et al. documented a 97% acceptance rate across hospital departments. Although slightly lower, our figure falls within the international range of 84–98%. Most accepted interventions in our dataset addressed dosing errors and duplicate therapies—problems that, if left unresolved, carry significant risks. The high acceptance underscores the pharmacist’s essential role in high-risk and dynamic settings. The analysis of intervention frequency by medication provided important insights into clinical priorities. Acetylsalicylic acid, the most frequently intervened drug (15.9%), highlights pharmacists’ role in balancing antiplatelet benefits with bleeding risks. This finding aligns with trends reported in Oman and the US, where antiplatelet and anticoagulant therapies are frequently employed in interventions. Enoxaparin (14.9%) further emphasizes the centrality of anticoagulation management, requiring precise dosing to minimize thrombosis and bleeding risks. Paracetamol (12.8%) also featured prominently, reflecting pharmacist vigilance in preventing hepatotoxicity, particularly in patients with liver impairment or alcohol use. These findings align with global reports showing frequent pharmacist interventions in analgesics to prevent overuse and related toxicity [16].

Ondansetron and omeprazole, accounting for nearly 19% of interventions, illustrate pharmacists’ efforts to optimize antiemetic and gastrointestinal therapies. Interventions with ondansetron often address QT prolongation risk, while those with omeprazole involve managing drug interactions, minimizing long-term overuse, and addressing cost considerations. Cholecalciferol and calcium carbonate interventions further highlight pharmacists’ role in optimizing therapy for bone health, ensuring safe dosing in osteoporosis and renal impairment. Similarly, frequent interventions with ibuprofen reflect pharmacist efforts to manage risks of bleeding and renal toxicity, especially in vulnerable populations. Amlodipine interventions demonstrate pharmacists’ contributions to optimizing antihypertensive therapy, while balancing side effects such as peripheral edema. Comparable findings were reported by Deyo et al., who showed that pharmacist-led interventions reduced high-risk medication use [17]. Behrens et al. also emphasized the role of pharmacists in managing anticoagulants and gastrointestinal drugs during transitions of care [18]. These patterns confirm that pharmacists consistently focus on high-risk, high-impact medications where the margin for error is narrow.

Analysis by specialty further underscores pharmacists’ pivotal role across healthcare settings. The Emergency Department (25.3% of interventions) was the leading site, reflecting its fast-paced environment where rapid prescribing decisions can lead to errors. Pharmacists in this setting resolve errors, provide consultations, and ensure safety during acute care. This finding aligns with Jacob et al., who demonstrated that pharmacist interventions in emergency settings reduce adverse drug events (ADEs) and associated costs [19]. Oncology clinics (9.8%) were the next most frequent, highlighting the role of pharmacists in tailoring chemotherapy, managing toxicities, and ensuring adherence to complex regimens. Arroyo et al. highlighted the necessity of pharmacists in minimizing toxicities and managing oncology drugs [20]. Male medical and hematology units followed closely (9.4% and 9.3%), reflecting the complexity of managing chronic diseases and anticoagulation. Cutler et al. showed that pharmacist interventions in hematology reduce errors and ADEs, aligning with our findings [21].

Medical clinics accounted for 8.8% of interventions, highlighting the essential role of pharmacists in primary care and chronic disease management. In these settings, pharmacists mitigate the risks of polypharmacy, improve adherence, and enhance patient education. Britt et al. documented substantial economic benefits from such interventions, including cost savings and better chronic disease control [22]. These comparisons underscore the importance of pharmacist involvement in both high-acuity and routine care settings, with significant direct clinical and economic implications. Categorizing interventions by condition further illustrates pharmacists’ targeted contributions. Hypoglycemia, for example, accounted for 34.9% of ICU admissions due to DRPs in our study, aligning with Abdelaziz et al., who reported its prevalence among diabetic patients [23]. Similarly, overdoses were a major contributor to DRPs, reflecting the need for careful pharmacological oversight. Interventions consistently reduced these risks. Ayhan et al. reported a 91.8% acceptance rate for interventions such as dose adjustment and therapy discontinuation, directly improving outcomes [24]. Özgan et al. demonstrated a 50% reduction in DRPs with pharmacist involvement in patients with renal dysfunction [25]. These findings affirm the pharmacist’s role in individualized dosing, particularly for vulnerable populations, and highlight how categorization facilitates more precise interventions.

Progress note documentation provided additional evidence of pharmacists’ systematic role in care optimization. In this study, 729 notes were recorded, with the majority originating from the pediatric ICU, followed by the surgical and medical ICUs and nephrology. This aligns with Althomali et al., who documented 404 ICU interventions over six months, most commonly addressing indications, with a 98.5% acceptance rate [26]. Alkhanbashi et al. reported 5310 interventions in a Saudi tertiary hospital, with more than half occurring in critical care and dosing adjustments being the most common category, achieving a 97% acceptance rate [27]. Hamadalneel et al. similarly emphasized safety-related interventions such as renal dose adjustments [28]. These findings align with our results, confirming that documentation is not merely an administrative task but is central to patient safety, accountability, and effective communication.

Collectively, these studies and the present findings underscore that consistent documentation of pharmacist interventions enables identification of trends, evaluation of effectiveness, and continuous improvement. Emphasis on dosing and safety interventions across multiple studies reflects a shared priority in minimizing preventable medication errors. Notably, the consistently high acceptance rates across diverse contexts (>96% in regional studies) highlight strong collaborative dynamics between pharmacists and physicians, reinforcing the integration of pharmacists into multidisciplinary teams.

Despite its strengths, this study has limitations. First, the retrospective design precludes the establishment of causal relationships between pharmacist interventions and outcomes. It may have resulted in missing or incomplete data, particularly in cases where documentation was inconsistent. Second, although the analysis was conducted using IBM SPSS Statistics, which provided robust descriptive analytics, the retrospective nature still restricted the scope for advanced inferential modeling. Third, variability in documentation practices may have led to underreporting or misclassification of interventions. Finally, the single-center design limits generalizability to institutions with different staffing models or CPS maturity.

## 5. Conclusions

This study highlights the crucial role of clinical pharmacists in identifying and resolving medication-related issues across various hospital settings. The findings reveal that dosing problems were the most encountered type of intervention. Our findings highlight that pharmacist interventions—particularly for dosing errors and high-risk medications—are key process measures that contribute to safer prescribing and support multidisciplinary care. Future research should focus on enhancing documentation practices, integrating CPS with advanced digital systems, and expanding its scope to further improve patient safety and therapeutic outcomes. These efforts will ensure that CPS continues to adapt to the evolving demands of healthcare and make meaningful contributions to patient care.

## Figures and Tables

**Table 1:** Type of Pharmacy Intervention.

Type of Intervention	Number	Percentage
Wrong Dose	29,584	77%
Medication Availability	2519	7%
Wrong Medication	1761	5%
Pharmacy Clarification	1243	3%
Drug Information	1237	3%
Duplicate therapy	878	2%
Dosing Regimen Error	609	2%
other	184	1%

**Table 2:** Pharmacy intervention by type medication.

Medication	Number of Interventions	Percentage
Acetylsalicylic Acid	4498	16%
Enoxaparin	4224	15%
Paracetamol	3619	13%
Ondansetron	2716	10%
Omeprazole Capsule	2660	9%
Cholecalciferol	2648	9%
Calcium Carbonate	2501	9%
Ibuprofen	2059	7%
amlodipine	1820	6%
Furosemide	1589	6%

**Table 3:** Frequency of intervention ordered by specialty.

Interventions by Medical Specialty	Number	Percentage
Emergency	11,050	25%
Oncology Clinic	4285	10%
Male Medical Unit	4119	9%
Hematology Clinic	4041	9%
Internal Medicine Clinic	3839	9%
Female Surgical Unit	3806	9%
Female Medical Unit	3288	8%
Pediatric Clinic	3160	7%
Male Surgical Unit	3108	7%
Hematology/Oncology Inpatient Unit	3001	7%

**Table 4:** Number of clinical pharmacist progress notes by specialty.

	Pediatric ICU	Nephrology	Medical ICU	Surgical ICU
Number of progress notes	324	97	123	185

**Table 5:** Number of clinical pharmacist progress notes by patient condition.

	Pediatric ICU	Nephrology	Medical ICU	Surgical ICU
Stable	181	66	49	76
Poor	0	0	11	28
Improvement	0	0	8	6
Deteriorating	0	0	0	3

## Data Availability

The original contributions presented in this study are included in the article. Further inquiries can be directed to the corresponding author.

## References

[1] Hepler C.D., Strand L.M. (1990). Opportunities and responsibilities in pharmaceutical care. Am. J. Hosp. Pharm..

[2] Bond C.A., Raehl C.L., Franke T. (2000). Clinical pharmacy services, hospital pharmacy staffing, and medication errors in United States hospitals. Pharmacother. J. Hum. Pharmacol. Drug Ther..

[3] Schumock G.T., Butler M.G., Meek P.D., Vermeulen L.C., Arondekar B.V., Bauman J.L. (2003). Evidence of the economic benefit of clinical pharmacy services: 1996–2000. Pharmacotherapy.

[4] Kaboli P.J., Hoth A.B., McClimon B.J., Schnipper J.L. (2006). Clinical pharmacists and inpatient medical care: A systematic review. Arch. Intern. Med..

[5] Kohn L.T., Corrigan J.M., Donaldson M.S. (2000). To Err Is Human: Building a Safer Health System.

[6] Mekonnen A.B., McLachlan A.J., Brien J.E. (2016). Effectiveness of pharmacist-led medication reconciliation programmes on clinical outcomes at hospital transitions: A systematic review and meta-analysis. BMJ Open.

[7] Chisholm-Burns M.A., Lee J.K., Spivey C.A., Slack M., Herrier R.N., Hall-Lipsy E., Graff Zivin J., Abraham I., Palmer J., Martin J. (2010). US pharmacists’ effect as team members on patient care: Systematic review and meta-analyses. Med. Care.

[8] Alomi Y.A., Al-Jarallah S.M., Al-Jazairi A.S., Al-Shaibani A.S. (2017). The national survey of hospital pharmacy practice at MOH hospitals in Saudi Arabia: Clinical pharmacy services. J. Pharm. Pr. Community Med..

[9] Alomi Y.A., Al-Doughan F., Al-Jarallah S.M., Ibrahim Y.A., Alragas A.M., Haidarah N.O.B. (2018). National survey of clinical pharmacy services in Saudi Arabia 2017–2018: Hospital pharmacy practice. Pharm. Pract..

[10] Pellegrino A.N., Martin M.T., Tilton J.J., Touchette D.R. (2009). Impact of clinical pharmacy services on health-related quality of life in patients with cardiovascular disease: A systematic review. Pharmacotherapy.

[11] Rivkin A., Yin H. (2011). Evaluation of the role of the critical care pharmacist in identifying and avoiding or minimizing significant drug-drug interactions in medical intensive care patients. J. Crit. Care..

[12] IBM Corp (2022). IBM SPSS Statistics for Windows, Version 29.0.

[13] Onatade F., Auyeung V., Scutt G., Wright J. (2021). Pharmacist-led interventions in UK hospitals: A focus on outcomes and prescriber acceptance. J. Clin. Pharm. Ther..

[14] Ylä-Rautio H., Siissalo S., Leikola S. (2020). Drug-related problems and pharmacy interventions in non-prescription medication, with a focus on high-risk over-the-counter medications. Int. J. Clin. Pharm..

[15] Baptista R., Williams M., Price J. (2023). Improving the impact of pharmacy interventions in hospitals. BMJ Open Qual..

[16] Al-Maqbali J.S., Taqi A., Al-Ajmi S., Al-Hamadani B., Al-Hamadani F., Bahram F., Al-Balushi K., Gamal S., Al-Lawati E., Al Siyabi B. (2022). The impacts of clinical pharmacists’ interventions on clinical significance and cost avoidance in a tertiary care university hospital in Oman: A retrospective analysis. Pharmacy.

[17] Deyo J.C., Smith B.H., Biola H., Ferry E.M., Orto V.K., Patel B., Stillwell T.K. (2020). Reducing high-risk medication use through pharmacist-led interventions in an outpatient setting. J. Am. Pharm. Assoc..

[18] Behrens A.R., Ha M.E., Addisu B. (2021). Assessment of inpatient pharmacists’ clinical interventions following implementation of a pharmacist intervention tracking tool. J. Pharm. Soc. Wis..

[19] Jacob S., Britt R.B., Bryan W.E., Hashem M.G., Hale J.C., Brown J.N. (2019). Economic outcomes associated with safety interventions by a pharmacist-adjudicated prior authorization consult service. J. Manag. Care Spec. Pharm..

[20] Arroyo J.P., Johnson D.C., Lewis J.B., Al Sheyyab A., King A., Danter M.R., McGrane S., Fessel J.P. (2018). Treatment of acute intoxication from inhaled 1,2-difluoroethane. Ann. Intern. Med..

[21] Cutler R.L., Fernandez-Llimos F., Frommer M., Benrimoj C., Garcia-Cardenas V. (2018). Economic impact of medication non-adherence by disease groups: A systematic review. BMJ Open.

[22] Jacob S., Britt R.B., Bryan W.E., Hashem M.G., Hale J.C., Brown J.N. (2019). Economic outcomes associated with pharmacist interventions. J. Manag. Care Spec. Pharm..

[23] Abdelaziz K., Abdelrahim M.E. (2015). Identification and categorization of drug-related problems on admission to an adult intensive care unit. Eur. J. Hosp. Pharm..

[24] Ayhan Y.E., Karakurt S., Sancar M. (2022). The effect of the clinical pharmacist in minimizing drug-related problems and related costs in the intensive care unit in Turkey: A non-randomized controlled study. J. Clin. Pharm. Ther..

[25] Özgan B., Ayhan Y.E., Apikoglu S., Karakurt S. (2024). Clinical pharmacist interventions in nutrition- and drug-related problems in critically ill patients with renal dysfunction: A non-randomized controlled study. Front. Med..

[26] Althomali A., Altowairqi A., Alghamdi A., Alotaibi M., Althubaiti A., Alqurashi A., Al Harbi A., Algarni M.A., Haseeb A., Elnaem M.H. (2022). Impact of clinical pharmacist intervention on clinical outcomes in the critical care unit, Taif City, Saudi Arabia: A retrospective study. Pharmacy.

[27] AlKhanbashi R.O., AlNoamy Y., Ghandorah R., Awan R.M., AlButi H. (2024). Assessment of clinical pharmacist interventions using a web-based application in a Saudi Arabian tertiary hospital. SAGE Open Med..

[28] Hamadalneel Y.B., Ahmed H.O. (2024). Impact of clinical pharmacist intervention in the intensive care unit, Wad Medani, Sudan: A cross-sectional, prospective study. Integr. Pharm. Res. Pract..

